# Unmet need for family planning and associated factors among currently married women in Hawella Tulla subcity, Hawassa, southern Ethiopia: community-based study

**DOI:** 10.1186/s40834-022-00212-w

**Published:** 2023-02-10

**Authors:** Abiyu Ayalew Assefa, Samson G. Selassie, Abebayehu Mesele, Henok Bekele Kebede, Anteneh Fikrie, Geleta Abera

**Affiliations:** 1 Department of Public Health, Hawassa College of health science, P.O.Box 84, Hawassa, Ethiopia; 2 Hawassa University Student Clinic, Hawassa, Ethiopia; 3 Departement of Public Health, Pharma College Hawassa Campus, PO.Box 67, Hawassa, Ethiopia; 4 School of Public Health, Institute of Health, Bule Hora University, PO. Box 144, Bule Hora, Ethiopia

**Keywords:** Factors, Family planning, Hawassa, Unmet need

## Abstract

**Background:**

The unmet need for family planning remains a major public health concern in developing countries, especially in sub-Saharan Africa. Similarly, in Ethiopia, the unmet need for family planning is considerably high. However information regarding associated factors of unmet need of family planning is limited, the study area in particular. Thus, this study was aimed at assessing unmet family planning and associated factors among currently married women in Hawella Tulla Subcity.

**Methods:**

A community based cross-sectional study was employed on 436 currently married women. Both bivariable and multivariable logistic regression model were used and having *P*-value of < 0.05 was considered as independently associated factors. Strength of association of the variable was described using adjusted odd ratios with their 95% confidence interval.

**Result:**

The overall unmet need for family planning among currently married women was found to be 18.1% (95% CI: 14.5%, 21.8%). Having age of below 18 years at first marriage AOR = 1.95 (95% CI: 1.14, 3.33), woman’s not attained formal education AOR = 2.23 (95% CI: 1.02, 4.84), women whose partner had non-supportive for family planning use AOR = 2.32 (95% CI: 1.35, 3.99) and women without media access AOR = 2.13 (95% CI: 1.19, 3.81) were significantly associated with increasing unmet need for family planning.

**Conclusions:**

Despite the presence of high family planning services coverage in the study area, the magnitude of unmet need for family planning is still reasonably high. Having age of below 18 years at first marriage, woman’s not attained formal education, women whose partner had non-supportive for family planning use and inavailability of media access in the house were found to be associated with high unmet need for family planning. Therefore, efforts are needed to empower women through education, avoiding early marriage and encouraging couple-based family planning interventions. Increasing media access is also advisable intervention.

## Background

Unmet need for family planning (FP) is an indicator that shows the proportion of sexually active women who either wish to delay the next birth (spacers) or who wish to stop childbirth (limiters) but are not using any method of contraception [1–3]. The unmet need for FP shows the gap between women’s reproductive intentions and their contraceptive behavior. In principle, this indicator may range from 0 to 100 [4].

The unmet need for FP is a major public health concern in developing countries, particularly in sub-Saharan Africa [5]. Around the world, an estimated 225 million women in developing countries who want to delay, space, or avoid becoming pregnant are not using effective methods of contraception, resulting in over 75 million unintended pregnancies every year [6], and these account for 84% of unintended pregnancies in developing countries [7]. In addition, Sub-Saharan Africa and Southern Asia account for 57% of women with an unmet need for modern contraception [8].

Despite having total fertility of 4.6 children per woman [9] and high family planning services coverage in almost all public health and private facilities of Ethiopia, only 41% of married Ethiopian women were using contraception in 2019 [10]. Similarly, in Ethiopia, the unmet need for family planning remains considerably high having a magnitude of 22% [9].

In Low- and Middle-Income Countries (LMICs), unintended pregnancies, unsafe abortions, and maternal deaths would drop by about two-thirds if all women in LMICs wanting to avoid pregnancy were to use modern contraceptives and all pregnant women were to receive care that meets international standards [11]. Furthermore, reducing the exposure of women to the risks of pregnancy, unintended, closely spaced pregnancies, infant, child mortality enhancing women’s education, employment, and productivity, improving the status of women in society were agreed benefits of family planning for women [12, 13].

Global and national initiatives have been tried to increase contraceptive uptake. Contraceptive prevalence and the unmet need for family planning are key indicators for measuring improvements in access to reproductive health as emphasized in the 2030 Agenda for Sustainable Development under target 3.7 [3, 14]. Family Planning 2020, a global partnership launched in 2012 aims to add 120 million new users of modern contraceptives in the world’s 69 poorest countries by 2020 [6]. As a member of the international community, the Federal ministry of health (FMOH) of Ethiopia has endorsed Family planning 2020 and updated its commitment by reinforcing family planning as a human right and currently working towards reducing the unmet need for family planning from 22% in 2016 to 10% by the end of 2020. Furthermore, the FMOH is dedicated to increasing the financing of FP services by continuing to allocate incrementally for its FP budget [15, 16].

Fear or experience of side effects, cultural or religious opposition, poor quality of available services, gender-based barriers, and infrequent sex were some of the reasons cited by women for not using family planning methods [17, 18]. Previous studies conducted in different parts of the world evidenced, the age of women [19], average monthly income [20], educational status of women [21], partner educational status [21], occupational status of women [22], and age at marriage of women [23] partner discussion [23], as determinants of unmet need for family planning.

The reasons behind the high unmet need for family planning remained unclear despite various government efforts and subsequent improvements in family planning service coverage in Ethiopia. It is for this reason that this study was undertaken to provide current information on the magnitude and determinants that influence the unmet need for FP in the study area.

Understanding issues related to the unmet need for family planning will inform policymakers, programmers, and other stakeholders to strengthen family planning and other health intervention programs to achieve international and national commitments which targeted maternal and under-five child mortality reduction. Similarly, no recent community-based assessment of the unmet need for FP has been published to the best knowledge of the authors.

Therefore, this study was aimed at assessing the magnitude of the unmet need for family planning and associated factors among currently married women in Southern Ethiopia.

## Methods

### Study area

The study was conducted in the Hawella sub-city of Hawassa city administration. Hawassa city is located 275 km south of Addis Ababa (the capital city of Ethiopia) on the Trans-African Highway 4 Cairo-Cape Town. The city is organized into eight sub-cities including; the Hawella sub-city. Hawella sub-city has 12 kebeles, out of which 11 are rural while the rest is urban kebele. The sub-city has an estimated total population of 145,445, of which 24,206 are females of childbearing age (15–49 years). Currently, one primary hospital, seven health centers, and 20 health posts are providing family planning services for the population.

### Study design and period

A community-based cross-sectional study was employed from June 30 to August 30, 2021.

### Source and study population

All currently married women (15–49 years) living in the Hawella sub-city were considered as the source population whereas all currently married women living in four randomly selected kebeles were the study population.

### Inclusion and exclusion criteria

All currently married women who lived in the area for greater than six months were included in the study while all currently married women who were seriously ill during data collection were excluded from the study.

### Sampling size determination and sampling technique

The sample size (n) required for this study was determined using single population proportion formula (n = (Zα/2)^2^ p (1-p)/d^2^)) by considering the following assumptions; the proportion of 23.1%, based on the prevalence of unmet need for family planning among married reproductive age women in Toke Kutaye district [24], 95% confidence interval, 5% margin of error, 10% non-response rate and design effect of 1.5. Accordingly, the final sample size calculated was found to be 450.

**Fig. 1:**
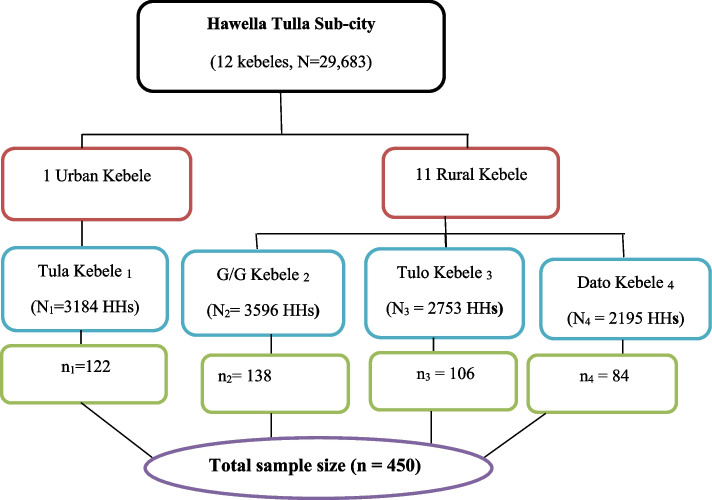
Schematic presentation of sampling procedure of currently married women in Hawella Tulla Sub-city, Hawassa, Southern Ethiopia, 2021

Multi-stage sampling technique was used to select kebeles and study participants. First, we stratified kebeles into urban and rural. Second, we selected four kebeles (one urban and three rural) randomly. Then, the total sample size was proportionally allocated to the selected urban and rural kebeles. Finally, from those selected kebeles, the study units (currently married women) were selected by using a systematic sampling technique. The sampling interval (K = 26) was determined by dividing the total number of households to the calculated sample size. After determining the first household to start with by lottery method, all currently married women at every 25th household were approached and informed consent was obtained to participate in this study. If there is no eligible women in the selected household, the immediate neighboring household was included. Furthermore, if respondents were not available at the time of visiting, another visit was arranged. In situations where more than one currently married women are available in a selected household, one woman was selected randomly by using the lottery method (Fig. 1).

### Study variables

The dependent variable for this study was the unmet need for family planning while the independent variables were sociodemographic factors (age of respondent, residence, respondent’s and husband’s occupational status, respondent’s and husband’s educational status, religion and ethnicity), socio-economic factors (average monthly income, media access), reproductive health-related factors (parity, current pregnancy, age at first marriage, age at firstborn), family planning related factors (contraceptive use and husband support for family planning use).

### Operational definition

*Unmet need for family planning*: In this study, the proportion of women who (a) are not pregnant and not postpartum amenorrhoeic and are considered fecund and want to postpone their next birth for 2 or more years or stop childbearing altogether but are not using a contraceptive method, or (b) have a mistimed or unwanted current pregnancy, or (c) are postpartum amenorrhoeic and their last birth in the last 2 years was mistimed or unwanted were considered to have an unmet need for family planning [9].

*Husband support*: in this study, we consider husband support if he is a volunteer, encouraging, and supportive of his wife in using family planning.

*Media access*: in this study, we considered “there is media access” if a woman says “yes” and “no media access” if she says “no” to the question” Do you have television or radio at your house”.

*Kebele*: The lowest government administrative hierarchy in Ethiopia.

### Data collection instrument and procedure

A structured questionnaire was used to collect relevant information from each currently married woman on socio-demographic factors, socio-economic factors, reproductive health factors and awareness-related factors, and the woman’s reason for unmet need for family planning. Most of the questions were closed-ended with pre-coded responses. The questionnaire was prepared by reviewing relevant literature and suited to this particular study [20–24]. Primary data was collected using an interviewer-administered questionnaire from currently married women. Three data collectors were recruited and two supervisors were assigned during the data collection process.

### Data quality assurance

The questionnaire was translated into the local language to Sidamigna and back to maintain its consistency. The training was given for 1 day for data collectors and supervisors. A pretest was done on 5% of the study sample size in an adjacent Kebele of the study area and necessary modifications were made to the questionnaire before the actual data collection. Supervision was made on daily basis during the data collection process so that any difficulties faced were resolved accordingly. Additionally, the collected data was swotted for completeness and consistency by supervisors and the principal investigator.

### Data processing and analysis

The collected data were coded, cleaned, and entered into Epi Data version 3.1 and exported to SPSS version 25 software for analysis. Descriptive statistics were used to describe study respondents by socio-demographic, socio-economic, reproductive health, and awareness-related factors using mean and standard deviation for continuous variables and frequency or percentage for categorical variables. Bivariable and multivariable logistic regression analyses were used to identify the influencing factors of the unmet need for family planning. All explanatory variables in bivariate analysis with a *p*-value of < 0.25 [25] were selected as candidate variables for multivariable logistic regression analysis. The crude and adjusted odd ratios together with their corresponding 95% confidence interval were calculated and interpreted accordingly. The strength of association was determined using the adjusted odds ratio (AOR) with their corresponding 95% confidence interval and a *p*-value of < 0.05 in the multivariable logistic regression were considered as a cut-off point to declare a significant association. The goodness-of-fit of the model was assessed using Hosmer and Lameshaw test with non-significant (*p*-value =0.344), representing a good-fit model.

## Results

### Socio-demographic and socio-economic characteristics of the respondents

A total of 436 currently married women agreed to participate in this study making a response rate of 96.9%. The mean (±SD) age of respondents was found to be 32.4 (±7.5) years. More than half (65.8%) of respondents were protestant by their religion. The majority, 275 (63.1%) of respondents were Sidama followed by Wolyita 49 (11.2%) in their ethnic background. One hundred fifty-seven (36.0%) of respondents had an average monthly income of greater or equal to three thousand Ethiopian birrs. With regard to educational status, 165 (37.8%) of respondents had no formal education. Similarly, 120 (27.5%) of respondents’ husbands had no formal education. One hundred twenty-nine (29.6%) of respondents were housewives by their occupational status. One hundred thirty-five (31%) respondents had no media access (Table 1).

**Table 1:** Distribution of socio-demographic characteristics among currently married women in Hawella Tula Sub-city, Hawassa, Southern Ethiopia, 2021

Variables	Frequency	Percent
**Age of respondent (in years)**		
≤ 24	51	11.7
25–34	214	49.1
≥ 35	171	39.2
**Educational status of the respondent**		
No formal education	165	37.8
Primary school	153	35.1
Secondary and above	118	27.1
**Occupational status of the respondents**		
Government employed	131	30.0
Self employed	150	34.4
House wife	129	29.6
Others^@^	26	6.0
**Average monthly income of the family (ETB)**		
≤ 1500	154	35.3
1501–3000	125	28.7
≥ 3001	157	36.0
**Religion of respondents**		
Protestant	287	65.8
Orthodox	92	21.1
Muslim	49	11.2
Others^@@^	8	1.8
**Place of residence**		
Urban	127	29.1
Rural	309	70.9
**Husband educational status**		
No formal education	120	27.5
Primary School	156	35.8
Secondary and Above	160	36.7
**Ethinicity**		
Sidama	275	63.1
Wolaita	49	11.2
Amhara	47	10.8
Oromo	36	8.3
others^@@@^	29	6.7
**Media access**		
Yes	301	69
No	135	31

^@^Others indicate: Daily labour (6), Student (8) and Farmer (12); ^@@^Others indicate: Catholic (5), Jehova witness (3); ^@@@^Others indicate: Gurage (13), Silte (16); ETB: Ethiopian birr

### Reproductive and family planning-related characteristics of respondents

From the total of 436 respondents, 173 (39.7%) got married for the first time before the age of eighteen years. Regarding current contraceptive use, near to half (49.5) of respondents used contraceptive methods at the time of data collection. The current study indicated that 72 (16.5%) of respondents were currently pregnant. Of all currently pregnant respondents, more than one-third (34.7%) of them were mistimed while only 7 (9.7%) of them were unwanted. More than half (56.7%) of respondents had two to four numbers of parity. Concerning husband support in the use of family planning, 247 (56.7%) of respondents had support from their husbands in the use of family planning (Table 2).

**Table 2:** Reproductive and family planning-related characteristics among currently married women in Hawella Tula Sub-city, Hawassa, Southern Ethiopia, 2021

Variables	Frequency	Percent
Age at first marriage (in years)		
< 18	173	39.7
> = 18	263	60.3
Current pregnant		
Yes	72	16.5
No	364	83.5
Pregnancy type		
Intended	40	55.6
Mistimed	25	34.7
Unwanted	7	9.7
Current contraceptive use		
Yes	216	49.5
No	220	50.5
Parity		
≤ 1	132	30.3
2–4	247	56.7
≥ 5	57	13.1
Husband support for family planning use		
Yes	247	56.7
No	189	43.3

### The magnitude of unmet need for family planning

**Fig. 2:**
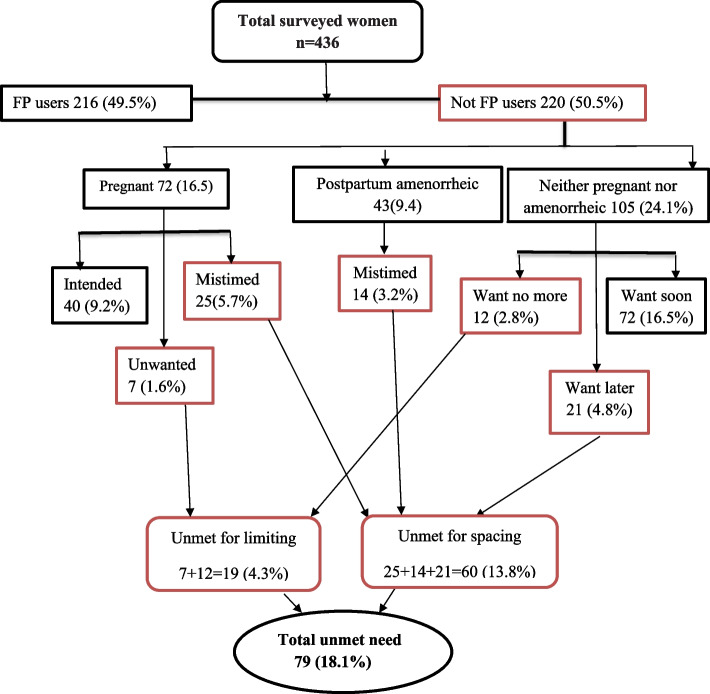
Pictorial illustration of calculated unmet need for family planning among currently married women in Hawella Tulla Sub-city, Hawassa, Southern Ethiopia, 2021

The unmet need for family planning among currently married women was found to be 79 (18.1%) [95% CI: 14.5%, 21.8%) of which, 60 (13.8%) were to delay while 19 (4.3%) of them were to limit the number of children (Fig. 2).

### Reasons for unmet need for family planning of respondents

**Fig. 3:**
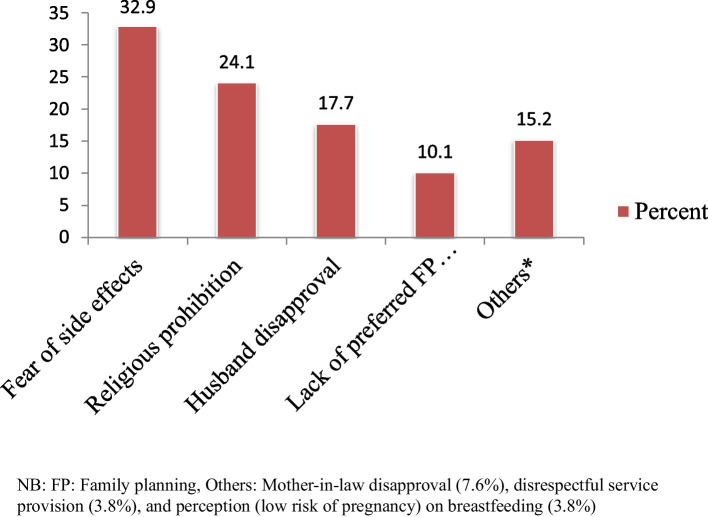
Reasons for unmet need for family planning among currently married women in Hawella Tulla Sub-city, Hawassa, Southern Ethiopia, 2021

The most mentioned reasons why women do not go in for use of family planning despite their desire to do so at the time of interview were fear of side effects (32.9%), religious prohibition (24.1), husband’s disapproval (17.7%), lack of preferred family planning method (10.1%) and others (15.2%) (Fig. 3).

### Factors associated with unmet need for family planning

Based on logistic regression results socio-demographic, reproductive, and family factors are assumed to be associated with the unmet need for family planning. Initially, bivariable logistic regression was conducted to select candidate variables or multivariate logistic regression analysis. Accordingly, eight variables (age of respondent, residence, woman’s educational status, husband’s educational status, media access, age at first marriage, parity, and husband support for family planning use) fulfilled the stated cutoff point and were selected as candidate variables. Then, candidate variables were entered into a multivariable logistic regression model to control the effects of potential confounders and declare independent factors of the unmet need for family planning. Hence, the final multivariable logistic regression analysis showed that age at first marriage, woman’s educational status, media access, and husband’s support for family planning use were found to be independent factors of the unmet need for family planning.

Women not attained formal education were more than two times more likely to have an unmet need for family planning compared with their counterparts (AOR = 2.23 (95% CI: 1.02, 4.84)). Likewise, women whose age at first marriage was less than 18 years were found nearly two times more likely to have an unmet need for family planning compared to those women whose age at first marriage was greater than 18 years (AOR = 1.95 (95% CI: 1.14, 3.33)). Furthermore, women who hadn’t support from their husbands in using family planning were more than two times more likely to have an unmet need for family planning (AOR = 2.32 (95% CI: 1.35, 3.99)) compared with their counterparts. Finally, women who hadn’t media access were found more than two times more likely to have an unmet need for family planning (AOR = 2.13 (95% CI: 1.19, 3.81)) compared to their counterparts (Table 3).

**Table 3:** Bi-variable and multivariable logistic regression of factors associated with unmet need for family planning among currently married women in Hawella Tulla Sub-city, Hawassa, Southern Ethiopia, 2021

Variables	Unmet need for FP		COR (95%CI)	AOR (95%CI)
	Yes	No		
**Age of respondent (in years)**				
<=24	13	38	1	1
25–34	34	180	0.55 (0.27, 1.14)*	0.57 (0.23, 1.42)
> = 35	32	139	0.67 (0.32, 1.41)	0.57 (0.19, 1.66)
**Educational status**				
No formal education	37	128	2.34 (1.18, 4.62)*	2.23 (1.02, 4.84)**
Primary school	29	124	1.89 (0.93, 3.82)*	1.55 (0.67, 3.57)
Secondary and above	13	105	1	1
**Husband educational status**				
No formal education	23	97	1.34 (0.72, 2.52)	0.65 (0.31, 1.35)
Primary School	32	124	1.46 (0.82, 2.62)*	1.25 (0.62, 2.53)
Secondary and Above	24	136	1	1
**Media Access**				
Yes	40	261	1	1
No	39	96	2.65 (1.61, 4.37)*	2.13 (1.19, 3.81)**
**Residence**				
Urban	28	99	1	1
Rural	51	258	0.70 (0.42, 1.17)*	0.84 (0.47, 1.50)
**Age at first marriage (in years)**				
< 18	45	128	2.37 (1.44, 3.89)*	1.95 (1.14, 3.33)**
≥ 18	34	229	1	1
**Parity**				
≤1	21	111	0.55 (0.26, 1.16)*	0.41 (0.14, 1.22)
2–4	41	206	0.53 (0.27, 1.05)*	0.57 (0.25, 1.30)
≥ 5	17	40	1	1
**Husband support for FP use**				
Yes	31	216	1	1
No	48	141	2.37 (1.44, 3.91)*	2.32 (1.35, 3.99)**

Hosmer–Lemeshow goodness-of-ft = 0.344

Abbreviations: COR, crude odds ratio; AOR, adjusted odds ratio; CI, confidence interval

NB. 1: reference, *Remained significant at P-Value < 0.25, **Remained significant at P-Value < 0.05

## Discussion

The overall aim of this study was to assess the magnitude of the unmet need for family planning and to identify associated factors among currently married women in Hawella Tulla Sub-city. The study found that 18.1% (95% CI: 14.5, 21.8%) of currently married women had an unmet need for family planning in the study area. Age at first marriage, woman’s educational status, media access, and husband support for family planning use have remained independent predictors of unmet need for family planning.

The magnitude of unmet need for family planning in this study was in line with the magnitude of 17.68% in Gambia [26], 20.79% in Mozambique [26], 21% in Zambia [27], 21% in Malawi [28], 15.8% in Eastern Sudan [29], 20.68% in East Africa [21], and 16.2% in Ethiopia [30]. On the other hand, the magnitude of the present study is low compared with previous magnitudes that reported 22.4% in Bangladesh [31], 24% in Indonesia [32], 39% in India [33], 23.5% in Pakistan [34], 32.6% in Saudi Arabia [35], 46.6% in Cameroon [36], 51.7% in Angola [37], and 23.1–34.6% in different parts of Ethiopia [24, 38–41]. The possible justification for this discrepancy might be due to differences in health services coverage, knowledge and attitudes towards family planning services, as socio-demographic and cultural factors. Besides this, variation in sample size might lead to substantial variation in the report.

However, studies conducted in Iran [42], reported a lower magnitude of unmet need for family planning (2.6%) compared with the magnitude of the current study. The possible explanation for this variation might be the difference in sociodemographic characteristics, particularly religious factors. Furthermore, the magnitude in the current study is far lower than national and international targets set to achieve 10% of unmet need for family planning in the year 2020 [15, 16].

Our study indicated that women educational status had a significant association with the unmet need for family planning. Women with no formal education were more than two times more likely to have an unmet need for family planning compared with women having secondary and above educational status. This finding was in agreement with previous studies conducted in Nepal [43], Pakistan [19, 34], Saudi Arabia [35], Malawi [28], East Africa [21], Burundi [20], and Ethiopia [23, 24, 41 and 44]. This might be explained by married women with no formal education who might not have decision-making power regarding their FP needs and lack economic independency as they are commonly unemployed. Additionally, educated women might have a better understanding of health messages and demand FP services that might increase their contraceptive use. Thus, giving more emphasis to less educated women so as to decrease the unmet need for family planning is advisable. On the contrary, studies done in Zambia [27] have shown that educational status had no significant association with the unmet need for family planning. The possible explanation might be due to differences in the sociodemographic characteristics of study participants.

The current study indicated that women married before the age of 18 years had two times higher chance of having an unmet need for family planning compared with their counterparts. This finding is consistent with other studies done in India [33], East Africa [21], and Ethiopia [23, 44, 45]. The possible reasons might be women under the age of 18 might not mature enough to make decisions upon FP need and lack of ability to cope with influences from husbands, mother-in-law, and the community at large. Hence, it is better to promote legal marriage, especially in rural areas.

Husband support was the other associated factor that affects the unmet need for FP among currently married women. Women with no support from husbands for family planning use had two times higher chance of having an unmet need for family planning compared with women who had husband support for family planning use. This finding is consistent with findings in Cameroon [36], Burkina Faso [46], Zambia [27], Sub-Saharan Africa [5], and Ethiopia [23, 24, 40, 47, 48]. The possible justification for this similarity might be due to the domination of the husband in decision-making compared with his wife. Therefore it is advisable to give priority to activities that encourage inter-spousal communication, involvement of men in counseling women for FP services, and empowering women in decision-making.

Furthermore, this study revealed that women who had no media access were two times more likely to have had an unmet need for FP than those women with media access. This result is comparable with other previous studies conducted in Pakistan [19, 34], Mozambique [26], Burundi [20], and Ethiopia [39, 40, 45]. The possible explanation might be due to the fact that media access can help to address different myths affecting contraceptive use by bringing behavioral change. Finally, despite the study including a more vulnerable group as study participants, it is not without weaknesses. So interpreting findings could require an understanding of the following limitations. First, there is no controlling mechanism of social desirability bias which might have affected participants’ responses. Second, as the study is cross-sectional, we could not establish a causal relationship between the independent and outcome variables. Lastly, since the study is focused only on currently married women, the identified factors might not work for all reproductive women.

## Conclusions

The study found that nearly one in five currently married women had an unmet need for FP; indicating a need for hard work in achieving the national target of unmet need for FP. Being under 18 years at first marriage, women with no formal education, women without partner support for FP use, and lack of media access were found to be associated with a high unmet need for FP. The increasing availability of family planning services only has no guarantee of addressing the unmet need for FP. Hence, programs should promote male involvement, encourage women’s education, tackle early marriage, and increase media coverage o family planning services. Since determinants of unmet needs are multifactorial, further research should consider using a qualitative approach to uncover factors related to lower unmet need for FP.

## Data Availability

The datasets generated and/or analyzed during this study are not available for online access. However, readers who wish to gain access to the data can write to the corresponding author Abiyu Ayalew Assefa at abiyman143@gmail.com.

## Abbreviations

AOR: adjusted odds ratio
COR: crude odds ratio
CI: Confidence interval
FMOH: Federal ministry of health
FP: Family planning
LMICs: Low- and Middle-Income Countries.
